# Use of virtual care near the end of life before and during the COVID-19 pandemic: A population-based cohort study

**DOI:** 10.1371/journal.pone.0313766

**Published:** 2025-01-08

**Authors:** Kieran L. Quinn, Thérèse A. Stukel, Allan Detsky, Hannah Chung, Mohammed Rashidul Anwar, Sacha Bhatia, James Downar, Vivian Hung, Sarina Isenberg, Allison Kurahashi, Douglas S. Lee, Nathan Stall, Peter Tanuseputro, Chaim M. Bell

**Affiliations:** 1 Department of Medicine, University of Toronto, Toronto, ON, Canada; 2 ICES, Toronto and Ottawa, ON, Canada; 3 Department of Medicine, Sinai Health System, Toronto, ON, Canada; 4 Temmy Latner Centre for Palliative Care, Sinai Health System, Toronto, ON, Canada; 5 Insitute of Health Policy, Management and Evaluation, University of Toronto, Toronto, ON, Canada; 6 Women’s College Hospital Institute for Health Systems Solutions and Virtual Care, Toronto, ON, Canada; 7 Division of Palliative Care, Dept of Medicine, University of Ottawa, Ottawa, ON, Canada; 8 Bruyere Research Institute, Ottawa, ON, Canada; 9 Queensland University of Technology School of Law, Brisbane City, Queensland, Australia; 10 Department of Family and Community Medicine, University of Toronto, Toronto, ON, Canada; 11 Peter Munk Cardiac Centre and the Ted Rogers Centre for Heart Research, University Health Network, Toronto, ON, Canada; 12 Ottawa Hospital Research Institute, Ottawa, ON, Canada; Global Health Neurology Lab / NSW Brain Clot Bank, NSW Health Pathology / Liverpool Hospital and South West Sydney Local Health District / Neurovascular Imaging Lab, Clinical Sciences Stream, Ingham Institute, AUSTRALIA

## Abstract

**Background and aims:**

The expanded use of virtual care may worsen pre-existing disparities in use and delivery of end-of-life care among certain groups of people. We measured the use of virtual care in the last three months of life before and after the introduction of virtual care fee codes that funded care delivery at the start of COVID-19 on March 14, 2020, and identified changes in the characteristics of people using it.

**Methods:**

We used linked clinical and administrative datasets to study use of virtual care in the last three months of life among 411,564 adults who died between January 25, 2018, and November 30, 2022. Modified Poisson regression was used to measure the association of the use of virtual care in the last three months of life with the pandemic study period and its association with each person- and physician-level factor.

**Results:**

14,261 people (8%) used virtual care in the last three months of life before the pandemic, and 161,000 people (69%) used it during the pandemic (relative risk [RR] 8.76; 95% CI 8.48–9.05). Several individual patient characteristics were associated with statistically significant increases in the use of virtual care after March 14, 2020 (following the introduction of virtual care fee codes), compared to before such as among older adults, ethnic minorities, multiple chronic comorbid health conditions and higher frailty groups.

**Conclusions:**

The introduction of new fee codes broadening technology and funding for end-of-life care at the start of pandemic combined with pandemic-related effects was associated with a substantial increase in the use of virtual care near the end of life among certain groups and a general leveling of pre-existing disparities in its use. Virtual end-of-life care delivery may strengthen person-centredness for individuals with limited ability to attend in-person appointments and by providers who may not have previously engaged in such care.

## Introduction

Virtual care involves the use of telemedicine and videoconferencing to deliver health services remotely [1]. On March 14, 2020, the Ontario Government introduced a set of reimbursable telephone and video-based physician fee codes to enable and fund the delivery of virtual care in response to COVID-19 pandemic-related restrictions and concerns about infection control for in-person clinical care, including virtual care near the end of life [2]. Prior to March 14, 2020, physicians were only reimbursed for delivery of virtual care near the end of life using a single telephone palliative care fee code up to twice per week, or a distinct set of video-based fee codes that required people to travel to an authorized virtual care centre.

For people nearing the end of life, virtual care has the potential to improve health outcomes, expand the pool of physicians who can deliver end-of-life care to address existing shortages, and increase access to care [1, 3–10]. These potential benefits are important given that fewer than one in four people receive end-of-life care at home [11–13], and there are global shortages of physicians to meet growing demand across health systems [14–16]. However, concerns have been raised regarding potential disparities in use of virtual care near the end of life among certain groups of individuals with poorer access to healthcare [17–30].

Little is known about whether virtual care can bridge pre-existing gaps or will further exacerbate disparities in use of community-based end-of-life care [25]. Consequently, the onset of the pandemic and the addition of new fee codes that were able to fund virtual end-of-life care delivery created a unique opportunity to study its use to inform its continued role in healthcare delivery in the post-pandemic era. Specifically, we addressed the potential impact of a “digital divide” whereby pre-existing disparities in use of palliative care may be worsened through increased use of digital technologies for care delivery. The objectives of this study were to measure the difference in the use of virtual care in the last three months of life before and after the introduction of new physician virtual care fee codes; and to identify changes in the types of people and physicians who used it. We defined virtual end-of-life care to be that which was provided in the last 3 months of life given that specific codes on virtual palliative care were not introduced in Ontario until July 2021. We hypothesized that the intervention would be associated with significant increases in the use of virtual care near the end of life, including across all individual characteristics of the people who used it, except among those who resided in rural regions where cellular service and bandwidth may be limited.

## Methods

This study is reported in accordance with guidelines for The Reporting of studies Conducted using Observational Routinely-collected health Data (RECORD) [31].

### Study design, setting and data sources

We conducted a population-based decedent cohort study in Ontario, Canada, using linked clinical and health administrative databases. The administrative datasets used in this study were linked using encoded identifiers at the person-level at ICES (formerly the Institute for Clinical and Evaluative Sciences) (S1 Table). These datasets are routinely used to conduct studies involving end-of-life care [14, 15, 32–35].

Ontario is Canada’s most populous province with over 13 million adults. Ontario is a geographically and ethnically diverse region, with most of its population living in urban centres, and a minority living in remote rural areas. All residents of Ontario have access to hospital care and physicians’ services, and those aged ≥ 65 years of age are provided universal prescription drug insurance coverage. The administrative datasets used in this study were linked using anonymized encoded identifiers at the person-level at ICES (formerly the Institute for Clinical and Evaluative Sciences). ICES is a prescribed entity under Ontario’s Personal Health Information Protection Act (PHIPA). Section 45 of PHIPA authorizes ICES to collect personal health information, without consent, for the purpose of analysis or compiling statistical information with respect to the management of, evaluation or monitoring of the allocation of resources to or planning for all or part of the health system. The use of the data in this project is authorized under section 45 and approved by ICES’ Privacy and Legal Office. The Mount Sinai Research Ethics Board waived the need for ethics review.

### Study cohort

Our cohort included all Ontario adults (age ≥18 years) in their last three months of life who died between January 25, 2018 (2 years prior to the first identified case of COVID-19 in Ontario, Canada) and November 30, 2022 (when specialized virtual care fee codes were phased out). The index date was three months before the person’s date of death. We excluded people who were missing data on age or sex, were <18 or >105 years of age (due to our intended study of adults and because of potential data quality issues for those aged >105 years), were non-residents of Ontario, were not eligible for the Ontario Health Insurance Plan (OHIP) for a period of ≥ three months in the year prior, did not have at least one health care encounter in last ten years, or were hospitalized during the entire period of follow-up as they would not be eligible to receive virtual care in the last three months of life. We also excluded people who resided in a nursing home due to their unique care environment and substantial disruption and variation in available care during the pandemic.

### Calendar date of the introduction of reimbursable physician virtual care fee codes (exposure)

Prior to March 14, 2020, physicians were reimbursed for the delivery of virtual care near the end of life by OHIP using a single telephone palliative care fee code or through the Ontario Telemedicine Network’s (OTN) distinct set of video-based fee codes. Notably, the telephone code was restricted to a maximum of use twice per week and only for the purposes of palliative care, and use of OTN required people and physicians to physically travel to an authorized virtual video conferencing care centre. On March 14, 2020, the Ontario Government introduced a set of reimbursable telephone and video-based physician fee codes to be used in any care setting (S2 Table). OHIP defines palliative care as “care provided to a terminally ill patient in the final year of life where the decision has been made that there will be no aggressive treatment of the underlying disease and care is to be directed to maintaining the comfort of the patient until death occurs.

People were assigned to the pre-pandemic or pandemic group based on their index date and date of their death. A person whose index and death dates were before March 14, 2020 was assigned to the pre-pandemic group. Conversely, a person whose index and death dates were on or after March 14, 2020 was assigned to the pandemic group. People who died <45 days before March 14, 2020 were assigned to the pre-pandemic group and people who died ≥45 days after were assigned to the pandemic group.

### Characteristics of people in the study cohort

We obtained demographic and clinical variables including age, sex, neighbourhood income quintile, indices of the Ontario Marginalization Index (ON-Marg)–a tool that combines a wide range of sociodemographic indicators into four distinct dimensions of marginalization (economic, ethno-racial, age-based and social marginalization), rural location of residence, surname-based ethnicity (Chinese, South Asian; all other ethnicities which were categorized as the “general population”) [36], chronic conditions [37–39], and the hospital frailty risk score [40] using a five-year look back period. We also recorded a person’s year of death, use of acute health care services and engagement with palliative care (using a distinct set of physician fee codes [14, 15, 32, 33]) in the one year before the study index date.

### Physicians characteristics

We identified the physician who was most responsible (MRP) for each person’s end-of-life care during the last three months of life using a modified sequential hierarchy from a recent study on MRP referral to palliative care [32]. We measured demographic variables including age, sex, rural location of practice, education, number of years in practice, specialty, status as a palliative care specialist and annual volume of visits. Physicians were deemed to be a palliative care specialist using a previously validated method with a sensitivity of 76.0% and specificity of 97.8% [41].

### Outcomes

The primary outcome was the use of virtual care in the last three months of life (i.e., virtual end-of-life care), which was measured using virtual care fee codes (S2 Table). Secondary outcomes included the number of physicians delivering virtual care in the last three months of life, and the use of specific virtual palliative care fee codes that were introduced on July 1, 2021 (S2 Table).

### Statistical analysis

We used modified Poisson regression that included person- and physician -level factors, clustered within physicians to measure the association between the use of virtual care in the last three months of life (outcome) with the pandemic study period (exposure; before vs. after March 14, 2020, when the pandemic virtual care fee codes were introduced). We did a complete case series analysis and those who were not assigned to a physician (and thereby missing physician characteristic information) were removed from the regression models. Adjusted models included all measured baseline characteristics.

To evaluate whether the health policy intervention differentially increased delivery of virtual care among certain people, we used descriptive statistics and modified Poisson regression to measure the association of each person- and physician-level factor (exposures) and the use of virtual care in the last three months of life (outcome) separately in subgroups with index dates before and after March 14, 2020. To corroborate the findings from the primary analysis, we conducted a separate analysis restricted to those who received virtual care in the last three months of life and used modified Poisson regression to measure the association between each person- and provider-level factor (exposures) and the pandemic period (outcome; pre- or during pandemic). This analysis examined if certain factors were more/less likely to be associated with receiving virtual care in the last 3 months of life in the pre- vs. pandemic period.

Balance across baseline person and physician characteristics at index date was assessed using standardised differences (SD), where SD ≤0·1 indicates good balance [42]. An alpha level <0.05 was considered statistically significant. All analyses were performed using SAS version 9·4 (SAS Institute, Cary, North Carolina, U.S.).

## Results

### Characteristics of the study cohort

**Fig 1:**
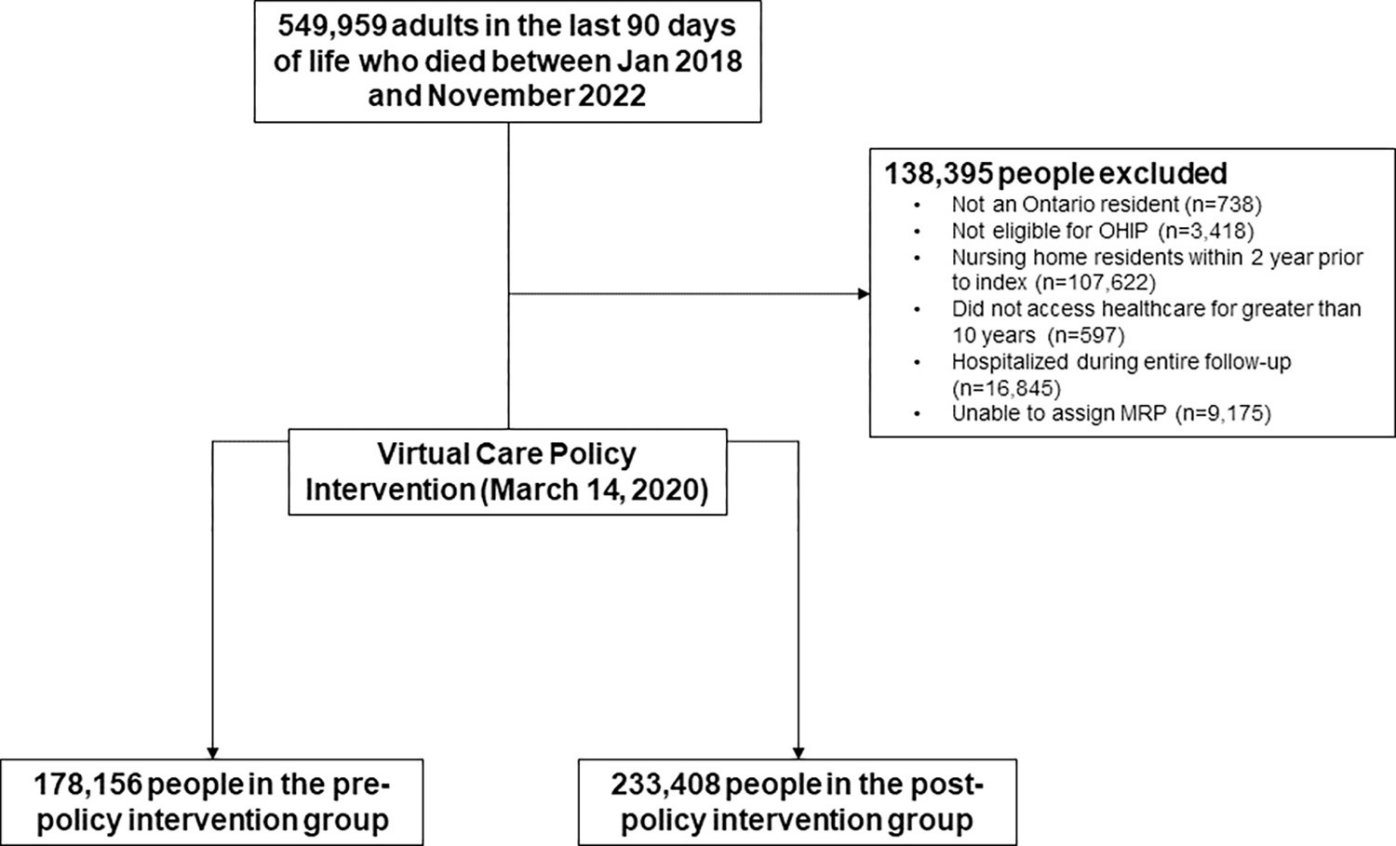
Construction of the study cohort.

The final study sample included 411,564 adults (median age 77 (IQR 66–87); 45% female) in their last three months of life (Fig 1). Prior to March 14, 2020, there were 19,698 unique physicians most responsible for delivering end-of-life care to 178,156 people who died. After March 14, there were 20,660 physicians most responsible for delivering end-of-life care to 233,408 people who died (16,995 physicians were assigned to people in both periods). There were 20,959 people whose index date was before March 14, 2020, but who died after. The characteristics of these people were similar each other and to the overall cohort. The baseline characteristics of all people and physicians were well balanced in the pre- and post-intervention period (S3 and S4 Tables). The individuals excluded from the study based on being hospitalization for the entirety of their last 3 months of life tended to be older, have higher frailty scores, have higher rates of cancer, dementia, and cardiovascular disease, and higher rates of prior healthcare use, regardless of the study period (S5 and S6 Tables).

### Changes in use of virtual care in the last three months of life

There were 14,261 people (8%) who received virtual care in the last three months of life before March 14, 2020, and 161,000 people (69%) who received it after. Following adjustment, the introduction of new fee codes for virtual care was significantly associated with increased use of virtual care in the last three months of life (RR 8.76; 95% CI 8.48–9.05, P<0.01). Among people who received virtual care after the introduction of specific virtual palliative care fee codes on July 1, 2021 (n = 86,465), there were 14,239 people (16%) who received at least one of this specific fee codes.

Most physicians did not deliver virtual care in the last three months of life to people in the pre-intervention period (mean number of most responsible physicians 0 [SD±4]), but many did in the post-intervention period (mean 6 [SD±11]). Seven percent (n = 1,403) of the physicians in the pre-pandemic period used virtual care in the last three months of life compared to 76% (n = 15,643) of the physicians in the pandemic period.

### Characteristics associated with changes in use of virtual care in the last three months of life

There was variation in the use of virtual care in the last three months of life across individual characteristics of the study cohort in the pandemic compared to the pre-pandemic period. During the pandemic, older adults, Chinese and South Asian ethnic minorities, those in the second neighbourhood income quintile, those with heart failure, dementia, diabetes, hypertension, chronic kidney disease, stroke, psychotic mental health disorders, and those who belonged to the highest frailty group were statistically significantly more likely to receive virtual care in the last three months of life. In contrast, its use statistically significantly decreased among younger adults, members of the general population, those living in rural areas, those with cancer, chronic obstructive pulmonary disorder and alcohol and substance uses disorders, and those who belonged to lower frailty groups (Table 1).

**Table 1:** Characteristics of adults who received virtual care in the last three months of life before and after the introduction of new reimbursable physician virtual care fee codes on March 14, 2020 and who died in Ontario between 2018 and 2022.

	Pre-Pandemic Group (n = 14,261)	Pandemic Group (n = 161,000)	StDiff
Use of virtual care in the last 3 months of life, RR (95% CI)	Referent	8.76 (8.48–9.05)	P<0.01
Age, n (%)			
18–29	223 (1.6%)	1,347 (0.8%)	0.07
30–39	389 (2.7%)	2,370 (1.5%)	0.09
40–49	593 (4.2%)	4,375 (2.7%)	0.08
50–59	1,604 (11.2%)	12,515 (7.8%)	0.12
60–69	3,008 (21.1%)	27,073 (16.8%)	0.11
70–79	3,615 (25.3%)	41,058 (25.5%)	0.0
80–89	3,384 (23.7%)	46,692 (29.0%)	0.12
90+	1,445 (10.1%)	25,570 (15.9%)	0.17
≥60 years old	11,452 (80.3%)	140,393 (87.2%)	0.19
Female Sex, n (%)	6,458 (45.3%)	74,212 (46.1%)	0.02
Neighborhood income quintile, n (%)			
1 (lowest)	3,632 (25.5%)	40,278 (25.0%)	0.01
2	3,047 (21.4%)	35,573 (22.1%)	0.02
3	2,718 (19.1%)	30,529 (19.0%)	0
4	2,462 (17.3%)	27,602 (17.1%)	0
5 (highest)	2,357 (16.5%)	26,513 (16.5%)	0
Missing	45 (0.3%)	505 (0.3%)	0
Ethnicity, n (%)			
Chinese	198 (1.4%)	4,818 (3.0%)	0.11
South Asian	208 (1.5%)	4,229 (2.6%)	0.14
General Population	13,855 (97.2%)	151,855 (94.3%)	0.08
Missing	0 (0.0%)	98 (0.1%)	0.03
Rural residence, n (%)	3,479 (24.4%)	18,210 (11.3%)	0.35
Chronic Conditions, n (%)			
Cancer	8,477 (59.4%)	82,382 (51.2%)	0.17
Chronic Heart failure	2,796 (19.6%)	38,175 (23.7%)	0.1
COPD	2,541 (17.8%)	24,608 (15.3%)	0.07
Dementia	1,289 (9.0%)	21,849 (13.6%)	0.14
Severe liver disease	247 (1.7%)	2,555 (1.6%)	0.01
Diabetes	4,430 (31.1%)	56,447 (35.1%)	0.09
Hypertension	7,038 (49.4%)	90,282 (56.1%)	0.13
Chronic Kidney Disease	2,731 (19.2%)	37,035 (23.0%)	0.09
Stroke	677 (4.7%)	8,509 (5.3%)	0.02
Psychotic disorder	213 (1.5%)	2,765 (1.7%)	0.02
Non-psychotic disorder	3,689 (25.9%)	40,862 (25.4%)	0.01
Alcohol and substance use disorder	1,345 (9.4%)	7,421 (4.6%)	0.19
Hospital frailty risk score, n (%)			
0	2,083 (14.6%)	19,070 (11.8%)	0.08
0·1–4·9	3,666 (25.7%)	36,712 (22.8%)	0.07
5·0–8·9	1,735 (12.2%)	19,715 (12.2%)	0.0
9·0 +	2,432 (17.1%)	32,100 (19.9%)	0.07
No prior hospitalizations	4,345 (30.5%)	53,403 (33.2%)	0.06
No. of ED visits not resulting in hospitalization, mean ± SD	1.74 ± 3.55	1.10 ± 2.23	-
No. of hospitalization episodes, mean ± SD	0.85 ± 1.32	0.72 ± 1.22	-
Receipt of palliative care in year prior to index, n (%)	1,520 (10.7%)	15,667 (9.7%)	-
Designated end-of-life, n (%)	2,315 (16.2%)	18,141 (11.3%)	-

COPD–Chronic Obstructive Pulmonary Disease; ED–Emergency department; StDiff–Standardized difference.

Note: Results for the Ontario Marginalization Index are not presented here because each of the 4 dimensions in the index are measured as quintiles and are too many to feasibly display.

**Fig 2:**
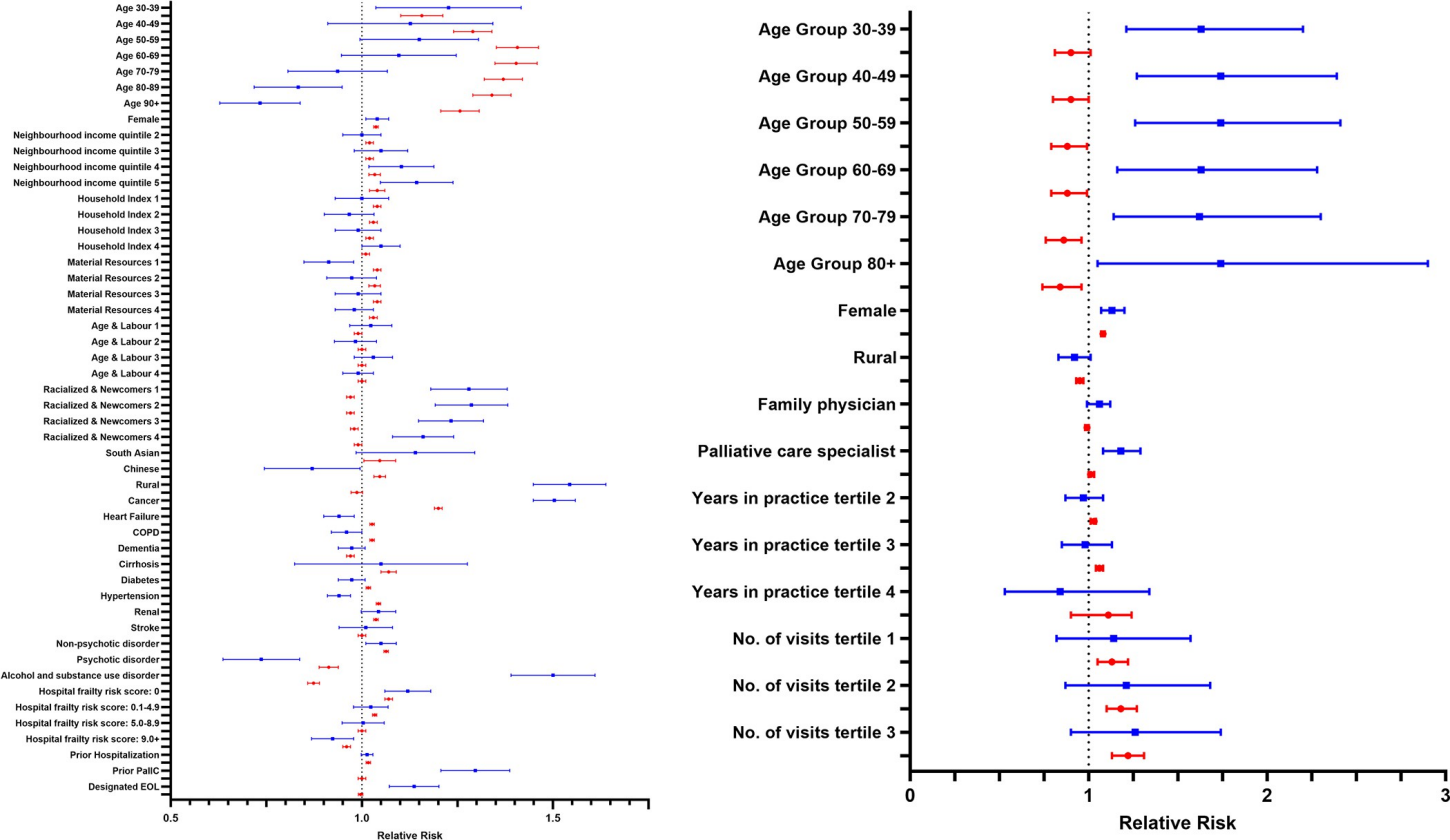
**A and B**. Forest plot of the adjusted relative risk of receiving virtual end-of-life care before (blue squares, pre-pandemic) and after (red circles, pandemic) the introduction of reimbursable virtual care physician fee codes on March 14, 2020 according to person-level **(Panel A)** and most responsible physician-level **(Panel B)** characteristics among adults in their last 3 months of life who died in Ontario between 2018 and 2022. An RR >1 indicates an increased and a RR < indicates a decreased likelihood of receiving virtual end-of-life care. Reference groups were age (vs. 18–29 years), female sex (vs. male), rural residence (vs. urban), neighbourhood income quintile (vs. 1^st^ quintile), household index quintile (vs. 5^th^ quintile), material resources index quintile (vs. 5^th^ quintile), age & labor index quintile (vs. 5^th^ quintile), racialized and newcomers index quintile (vs. 5^th^ quintile), surname-based ethnicity (vs. general population), chronic conditions (yes vs. no), hospital frailty risk score (vs. not hospitalized), prior hospitalization/palliative care/end-of-life designation (yes vs. no). COPD–chronic obstructive pulmonary disease; PallC–palliative care; EOL–end-of-life.

Using the patient visit as the unit of analysis, the use of virtual care in the last three months of life during the pandemic, compared to before statistically significantly increased among older physicians, males, those with more years of practice, family physicians, international medical graduates, and those with higher annual volumes of patient visits (Table 2). After adjustment for baseline characteristics, there was a general leveling of pre-existing disparities in its use across individual patient and physician characteristics in the pandemic, compared to pre-pandemic period (Fig 2A and 2B).

**Table 2:** Characteristics of most responsible physicians who delivered virtual end-of-life care to people in their last 3 months of life before and after the introduction of new reimbursable physician virtual care fee codes on March 14, 2020 and who died in Ontario between 2018 and 2022. Results are reported using the patient visit as the unit of analysis.

	Pre-Pandemic Group (N = 14,261)	Pandemic Group (N = 161,000)	StDiff
Age Group, n (%)			
≤29 years	189 (1.3%)	150 (0.1%)	0.15
30–39 years	3,820 (26.8%)	38,588 (24.0%)	0.06
40–49 years	3,908 (27.4%)	42,971 (26.7%)	0.02
50–59 years	3,665 (25.7%)	42,312 (26.3%)	0.01
60–69 years	2,240 (15.7%)	28,738 (17.8%)	0.06
70–79 years	414 (2.9%)	7,647 (4.7%)	0.1
80+ years	25 (0.2%)	594 (0.4%)	0.04
Female sex, n (%)	5,578 (39.1%)	59,939 (37.2%)	0.04
Rural practice, n (%)	1,512 (10.6%)	9,121 (5.7%)	0.18
Years in practice (tertiles), n (%)			
1	5,297 (37.1%)	51,700 (32.1%)	0.11
2	4,810 (33.7%)	53,870 (33.5%)	0.01
3	4,075 (28.6%)	55,210 (34.3%)	0.12
Missing	79 (0.6%)	220 (0.1%)	0.07
Family physician, n (%)	9,753 (68.4%)	115,357 (71.7%)	0.07
Palliative care specialist, n (%)	3,784 (26.5%)	38,136 (23.7%)	0.07
Medical Education			
Canadian graduate	9,376 (65.7%)	94,969 (59.0%)	0.14
I nternational graduate	2,592 (18.2%)	35,070 (21.8%)	0.09
Missing	2,293 (16.1%)	30,961 (19.2%)	0.08
No. of unique care visits during prior calendar year (tertiles), n (%)			
1	4,948 (34.7%)	50,612 (31.4%)	0.07
2	4,837 (33.9%)	53,555 (33.3%)	0.01
3	4,345 (30.5%)	56,095 (34.8%)	0.09
Missing	131 (0.9%)	738 (0.5%)	0.06

StDiff–Standardized difference.

## Discussion

This population-based cohort study demonstrated that broadening technology and funding for provision of end-of-life care during the pandemic and subsequent lock-downs due to COVID-19 was associated with substantial increases in overall use, evolving relationships with potential leveling of pre-existing demographic disparities in use among subgroups of individuals, and recruitment of new physicians to deliver virtual care in the last three months of life.

Our overall findings contrast with prior concerns that broader use of virtual care may worsen disparities in access near the end-of-life, which is novel [9, 19–21, 24, 28–30]. We found that prior to the pandemic, specific subgroups of potentially vulnerable people, such as older adults, frail individuals, those with certain chronic conditions, and those with underlying mental health disorders were less likely to use virtual care in the last three months of life, compared to during the pandemic period. However, many of these relationships and potential disparities were attenuated or eliminated following the introduction of virtual care fee codes at the onset of the pandemic. Still, some groups continued to experience relative decreases in use during the pandemic compared to prior, including those living in rural areas, where authorized virtual care centres may be inconvenient to access, or cellular phone service may be limited.

It will become increasingly essential to demonstrate the value of virtual care as a delivery model to the people who use it and the payers who reimburse it to maintain ongoing coverage on a system level [43]. The increase in use of virtual care by in the last three months of life during the pandemic may be a result of patient’s comfort and ability to use the telephone, lack of restrictions on physician use of fee codes, patient and physician concerns over infection control during in-person visits, or potentially a lack of patient choice. The vulnerability of people nearing the end-of-life–particularly during the pandemic–may have also driven increases in the use of virtual end-of-life care, especially during periods where complete lock-downs were not in effect. Alternatively, the enabling and funding of virtual care delivery may have more directly affected physicians and their practice behavior, offsetting some of the barriers that were predicted to occur resulting from personal, sociocultural, geographic, and technological factors [9, 19–21, 24, 28–30]. Our findings also support prior studies that suggested the use of virtual care may allow physicians to see more people and improve the overall capacity of health systems to deliver end-of-life care [44, 45].

Our study aligns with prior research that demonstrated substantial impacts of health policy interventions–in addition to the effects of the pandemic–on inducing changes in care delivery, including virtual care [2, 46, 47]. It is unclear if the observed nearly nine-fold increase in use of virtual care in the last three months of life is relatively low compared to increases found in other healthcare settings and populations of people not at end-of-life. In March 2020, the US Centers for Medicare & Medicaid Services expanded virtual care for Medicare beneficiaries using a policy intervention that authorized waivers allowing for a variety of virtual care visits. These visits increased nearly 62-fold from approximately 840,000 in 2019, to 52·7 million in 2020 and 92% of virtual visits in 2020 occurred in person’s homes [46]. A recent population-based cohort study in Ontario reported virtual care delivery increased following the introduction of specific virtual care fee codes from 1·6% of total ambulatory visits in the second quarter of 2019 to 70·6% in the second quarter of 2020 [2]. In a separate population-based cohort study of primary care in Ontario, 71% of all primary care visits were delivered virtually following the introduction of specific virtual care fee codes [48]. These relatively lower changes in use may be related to the intensity of care needs at the end-of-life and the need for more in-person visits to properly address them. Future research will be needed to address additional domains of care quality beyond access, including evaluation of patient and physician preferences for virtual versus in-person end-of-life care, and patient/caregiver reported outcomes such as symptoms, quality of life, satisfaction, and goal-concordance.

### Limitations

The observational nature of our study in the context of substantial changes in healthcare delivery and differences in the ways in which virtual care was delivered before and during the pandemic limits conclusions about the causative effects of a health policy intervention on the use and disparities in access to virtual care in the last three months of life. These include COVID-19 pandemic-related restrictions outside one’s home and limitations on in-person clinical care resulting in changes to community-based healthcare delivery. At the very least, the provision of new fee codes helped enable the observed changes in virtual care in the last three months of life during the midst of a pandemic and may help catalyze further transformation in healthcare delivery. Second, the present study examined the delivery of virtual care in the last three months of life without the ability to identify cause of death, and not virtual palliative care specifically. Still, we found that a substantial minority of the cohort received at least one specific virtual palliative care visit after these codes were introduced later in the pandemic. Further, some of the indications for use of the codes in the pre- and pandemic period (e.g., psychotherapy) limited the ability to examine potential differences in the types of virtual services most utilized in the pre-, compared to pandemic period. Third, we did not measure the associated effects of virtual care in the last three months of life on other important end-of-life outcomes such as quality of life, symptoms, and healthcare use. The significant increase in use of virtual care does not fully account for the quality of virtual care provided or associated patient outcomes. A recent study examined some of these important issues, demonstrating an association of virtual care at the end-of-life with higher rates of emergency room visits and hospitalizations [49]. Fourth, our study was limited to measures of physician-delivered end-of-life care. It is increasingly recognized that delivery of high-quality end-of-life care involves an interdisciplinary team involving multiple types of healthcare physicians who services are not routinely captured in administrative datasets. Fifth, our study may not be generalizable to people residing in nursing home facilities since they were excluded from this study due to their unique care environment and substantial disruption and variation in available care during the pandemic. Sixth, we were unable to measure the views, attitudes and acceptability of patients, caregivers and healthcare providers toward the use of virtual end-of-life care. A comparative evaluation of the causal effects of virtual end-of-life care on disparities in access and the provision of high-quality palliative care in the post-pandemic era bears further study.

## Conclusions

The introduction of new fee codes broadening technology and funding for end-of-life care at the start of pandemic combined with pandemic-related effects was associated with a substantial increase in the use of virtual care near the end of life among certain groups and a general leveling of pre-existing disparities in its use. Virtual end-of-life care delivery may strengthen person-centredness for individuals with limited ability to attend in-person appointments and by providers who may not have previously engaged in such care.

## Supporting information

S1 Table Description of linked health administrative datasets. (DOCX)

S2 Table List of virtual care fee codes according to pandemic time periods. (DOCX)

S3 Table Baseline characteristics of the study cohort. (DOCX)

S4 Table Baseline characteristics of most responsible providers. (DOCX)

S5 Table Comparison of baseline characteristics of people who were included in the study to those who were excluded from the study based on their being hospitalized for the entire duration of the study period. (DOCX)

S6 Table Comparison of baseline characteristics of people before and after the introduction of new reimbursable physician virtual care fee codes on March 14, 2020 who were included in the study to those who were excluded from the study based on their being hospitalized for the entire duration of the study period. (DOCX)

S7 Table Dataset creation and analysis plan. (DOCX)
