# Supplementary material for: Use of virtual care near the end of life before and during the COVID-19 pandemic: A population-based cohort study
Source: PLoS One. 2025 Jan 8;20(1):e0313766. doi: 10.1371/journal.pone.0313766 (PMC11709317; doi:10.1371/journal.pone.0313766)
Supplement: S1 Table — (DOCX) [file pone.0313766.s001.docx]

**S1 Table – Description of linked health administrative datasets**

| **Database** | **Description** |
| --- | --- |
|  |  |
| Canadian Institute for Health Information Discharge Abstract Database (CIHI-DAD) | Contains detailed diagnostic and procedural information for all hospital admissions in Canada.  DAD records have been demonstrated to have excellent agreement (over 99%) for demographic and administrative data. Regarding diagnoses, median agreement between original DAD records and re-abstracted records for the 50 most common most responsible diagnoses was noted to be 81% (Sensitivity 82%; Specificity 82%). The corresponding median agreement for the 50 most frequently performed surgical procedures was 92% (sensitivity 95%, positive predictive value 91%).^1^ |
|  |  |
| Continuing Care Reporting System Long-Term Care (CCRS-LTC) | Contains demographic, administrative, clinical and resource utilization information on patients who receive continuing care services in hospitals or long-term care (LTC) homes in Canada. The long-term care dataset is generated from the Individual Assessment Instrument Minimum Data Set 2.0, a mandatory comprehensive, standardized and validated instrument for evaluating the needs, strengths, and preferences of elderly adults residing in nursing homes and receiving home care, contains detailed information on the functional status of these people.^2^ Full assessments are completed on admission or referral, at quarterly intervals and following any significant health status change. |
|  |  |
| Home Care Database (HCD) | Contains patient-level data on government-funded home and community services. |
|  |  |
| National Ambulatory Care Reporting System (NACRS) | Reports demographic, administrative, clinical and service-specific data for Emergency Department visits. |
|  |  |
| National Rehabilitation Reporting System (NRS) | Contains patient data collected from participating adult inpatient rehabilitation facilities and programs across Canada |
|  |  |
| Ontario Congestive Heart Failure (CHF) | Contains all Ontario individuals with CHF identified since 1991.  A diagnosis of HF was identified by the presence of one hospital record or physician claim, followed by a second record from either source within 1 year. This method has been previously validated with a sensitivity of 84.8% and a specificity of 97.0%.^3^ |
|  |  |
| Ontario Drug Benefit (ODB) | Provides individual prescription records including all prescriptions dispensed to Ontario residents aged 65 years and older. Each medication claim has an associated prescriber identifier which indicates the health practitioner who wrote the prescription.  An audit of 5,155 randomly selected prescriptions dispensed from 50 Ontario pharmacies determined that the ODB had an error rate of 0.7% and none of the pharmacy characteristics examined (locations, owner affiliation, productivity) were associated with coding errors.^4^ |
|  |  |
| Ontario Health Insurance Plan (OHIP) | Identifies physician billing claims and specialty on all services provided by fee-for-service physicians in Ontario. |
|  |  |
| Ontario Mental Health Reporting System (OMHRS) | Documents data on patients in adult designated inpatient mental health beds.  This includes beds in General, Provincial Psychiatric, and Specialty Psychiatric facilities. |
|  |  |
| Office of the Registrar General – Deaths (ORGD)  Registered Persons Database (RPDB) | An annual dataset containing information on all deaths registered in Ontario starting on January 1 1990 that includes the cause of death as indicated on their death certificate.  Registry of all Ontarians eligible to receive insured health services in the province and contains detailed demographic information as well as the Local Health Integration Networks (LHIN), which defines Ontario 14 regional areas within which people received most of their hospital care from local hospitals. The RPDB also provides information on the date and location of death for all individuals in Ontario. |
|  |  |
| Same Day Surgery (SDS) | Contains patient-level data for day surgery institutions in Ontario. Every record corresponds to one same-day surgery or procedure stay |

1. Juurlink DN, Preyra C, Croxford R, et al. Canadian Institute for Health Information Discharge Abstract Database: A Validation Study.; 2006.

2. Mor V. A comprehensive clinical assessment tool to inform policy and practice: applications of the minimum data set. Medical care. 2004;42(4 Suppl):III50-9.

3. Schultz SE, Rothwell DM, Chen Z, Tu K. Identifying cases of congestive heart failure from administrative data: a validation study using primary care patient records. Chronic diseases and injuries in Canada. 2013;33(3):160-166.

4. Levy AR, O’Brien BJ, Sellors C, Grootendorst P, Willison D. Coding accuracy of administrative drug claims in the Ontario Drug Benefit database. The Canadian journal of clinical pharmacology = Journal canadien de pharmacologie clinique. 2003;10(2):67-71.
