# Supplementary material for: Use of virtual care near the end of life before and during the COVID-19 pandemic: A population-based cohort study
Source: PLoS One. 2025 Jan 8;20(1):e0313766. doi: 10.1371/journal.pone.0313766 (PMC11709317; doi:10.1371/journal.pone.0313766)
Supplement: S2 Table — (DOCX) [file pone.0313766.s002.docx]

**S2 Table – List of virtual care fee codes according to pandemic time periods.**

**Pre-Pandemic Fee Codes**

B099A Tracking Code

B100A First Telemedicine Patient Encounter premium

B101A First Cancelled/Missed Telemedicine Patient Encounter premium

B102A First Technical Difficulties Abandoned Patient Encounter premium

B200A Subsequent Telemedicine Patient Encounter premium

B201A Subsequent Missed/Cancelled Telemedicine Patient Encounter premium

B202A Subsequent Technical Difficulties Abandoned Patient Encounter premium

G511A Telephone management regarding a patient receiving palliative care at home

**Pandemic Fee Codes**

K080A Minor assessment of a patient by telephone or video or advice or information by telephone or video to a patient’s representative regarding health maintenance, diagnosis, treatment and/or prognosis.

K081A *a.* Intermediate assessment of a patient by telephone or video, or advice or information by telephone or video to a patient’s representative regarding health maintenance, diagnosis, treatment and/or prognosis, if the service lasts a minimum of 10 minutes; or

*b.* Psychotherapy, psychiatric or primary mental health care, counselling or interview conducted by telephone or video, if the service lasts a minimum of 10 minutes.

K082A Psychotherapy, psychiatric or primary mental health care, counselling or

interview conducted by telephone or video per unit (unit means half hour or major part thereof).

K083A Specialist Consultations and Visits by telephone or video.

B203A Synchronous video visits with a patient in the home or another location of their choice (i.e. the patient is not at a patient host site).

G511A Telephone management regarding a patient receiving palliative care at home
