# Supplementary material for: Use of virtual care near the end of life before and during the COVID-19 pandemic: A population-based cohort study
Source: PLoS One. 2025 Jan 8;20(1):e0313766. doi: 10.1371/journal.pone.0313766 (PMC11709317; doi:10.1371/journal.pone.0313766)
Supplement: S3 Table — (DOCX) [file pone.0313766.s003.docx]

**S3 Table - Baseline characteristics of people in their last 3 months of life before and after the introduction of new reimbursable physician virtual care fee codes on March 14, 2020 and who died in Ontario between 2018 and 2022.**

|  | **Pre-Pandemic Group (n=178,156)** | **Pandemic**  **Group**  **(n=233,408)** | **Standardized Difference** |
| --- | --- | --- | --- |
| Age |  |  |  |
| Median (IQR) | 77 (66-86) | 77 (66-87) | 0.02 |
| 18-29 | 2,669 (1.5%) | 3,533 (1.5%) | 0 |
| 30-39 | 3,483 (2.0%) | 5,324 (2.3%) | 0.02 |
| 40-49 | 5,906 (3.3%) | 7,830 (3.4%) | 0 |
| 50-59 | 15,784 (8.9%) | 19,126 (8.2%) | 0.02 |
| 60-69 | 30,009 (16.8%) | 38,133 (16.3%) | 0.01 |
| 70-79 | 41,819 (23.5%) | 55,264 (23.7%) | 0 |
| 80-89 | 50,585 (28.4%) | 64,738 (27.7%) | 0.01 |
| 90+ | 27,901 (15.7%) | 39,460 (16.9%) | 0.03 |
| Female Sex, n (%) | 80,436 (45.1%) | 104,836 (44.9%) | 0 |
| Neighborhood income quintile, n (%) |  |  |  |
| Missing | 661 (0.4%) | 928 (0.4%) | 0 |
| 1 | 46,511 (26.1%) | 62,559 (26.8%) | 0.02 |
| 2 | 39,270 (22.0%) | 51,727 (22.2%) | 0 |
| 3 | 33,716 (18.9%) | 43,637 (18.7%) | 0.01 |
| 4 | 29,731 (16.7%) | 38,410 (16.5%) | 0.01 |
| 5 | 28,267 (15.9%) | 36,147 (15.5%) | 0.01 |
| Ethnicity, n(%) |  |  |  |
| Missing | 0 (0.0%) | 149 (0.1%) | 0.04 |
| Chinese | 4,333 (2.4%) | 6,330 (2.7%) | 0.02 |
| South Asian | 3,497 (2.0%) | 5,381 (2.3%) | 0.02 |
| General Population | 170,326 (95.6%) | 221,548 (94.9%) | 0.03 |
| Rural Residence, n (%) | 23,365 (13.1%) | 29,590 (12.7%) | 0.01 |
| Chronic Conditions, n (%) |  |  |  |
| Cancer | 83,046 (46.6%) | 101,394 (43.4%) | 0.06 |
| Heart failure | 40,940 (23.0%) | 51,015 (21.9%) | 0.03 |
| COPD | 29,626 (16.6%) | 32,903 (14.1%) | 0.07 |
| Dementia | 22,751 (12.8%) | 32,612 (14.0%) | 0.04 |
| Severe liver disease | 2,294 (1.3%) | 3,109 (1.3%) | 0 |
| Diabetes | 57,198 (32.1%) | 76,718 (32.9%) | 0.02 |
| Hypertension | 96,323 (54.1%) | 122,121 (52.3%) | 0.03 |
| End-stage renal disease | 37,210 (20.9%) | 48,906 (21.0%) | 0 |
| Stroke | 9,637 (5.4%) | 11,888 (5.1%) | 0.01 |
| Psychotic disorder | 3,237 (1.8%) | 4,942 (2.1%) | 0.02 |
| Non-psychotic disorder | 42,148 (23.7%) | 56,683 (24.3%) | 0.01 |
| Alcohol and substance use  disorder | 9,975 (5.6%) | 14,607 (6.3%) | 0.03 |
| Hospital frailty risk score, n (%) |  |  |  |
| 0 | 20,253 (11.4%) | 24,777 (10.6%) | 0.02 |
| 0.1 - 4.9 | 40,314 (22.6%) | 49,468 (21.2%) | 0.03 |
| 5.0 - 8.9 | 20,945 (11.8%) | 27,096 (11.6%) | 0 |
| 9.0 + | 34,206 (19.2%) | 45,076 (19.3%) | 0 |
| No prior hospitalizations | 62,438 (35.0%) | 86,991 (37.3%) | 0.05 |
| No. of ED visits not resulting in hospitalization, mean ± SD | 1.22 ± 2.74 | 1.05 ± 2.43 | 0.07 |
| No. of hospitalization episodes, mean ± SD | 0.71 ± 1.24 | 0.63 ± 1.17 | 0.07 |
| Receipt of palliative care in year prior to index, n (%) | 11,969 (6.7%) | 18,783 (8.0%) | 0.05 |
| Designated end-of-life, n (%) | 17,615 (9.9%) | 21,885 (9.4%) | 0.02 |

COPD – Chronic Obstructive Pulmonary Disease; ED – Emergency department
