# Supplementary material for: Use of virtual care near the end of life before and during the COVID-19 pandemic: A population-based cohort study
Source: PLoS One. 2025 Jan 8;20(1):e0313766. doi: 10.1371/journal.pone.0313766 (PMC11709317; doi:10.1371/journal.pone.0313766)
Supplement: S4 Table — (DOCX) [file pone.0313766.s004.docx]

**S4 Table – Baseline characteristics of most responsible physicians who delivered end-of-life care to people in their last 3 months of life before and after the introduction of new reimbursable physician virtual care fee codes on March 14, 2020 and who died in Ontario between 2018 and 2022.**

|  | **Pre-Pandemic Group**  **(N=19,698)** | **Pandemic Group**  **(N=20,660)** | **Standardized Difference** |
| --- | --- | --- | --- |
| Age, median (IQR) | 48 (39-59) | 49 (40-59) | 0.04 |
| Female sex, n (%) | 7,517 (38.2%) | 8,285 (40.1%) | 0.04 |
| Rural practice, n (%) | 1,140 (5.8%) | 1,109 (5.4%) | 0.02 |
| Education |  |  |  |
| Canadian graduate | 11,884 (60.3%) | 11,669 (56.5%) | 0.08 |
| International graduate | 4,477 (22.7%) | 4,358 (21.1%) | 0.04 |
| Missing | 3,337 (16.9%) | 4,633 (22.4%) | 0.14 |
| Years in practice, median (IQR) | 23 (12-33) | 23 (13-33) | 0.02 |
| Family physician, n (%) | 11,499 (58.4%) | 11,855 (57.4%) | 0.02 |
| Palliative care specialist, n (%) | 719 (3.7%) | 841 (4.1%) | 0.02 |
| No. of unique care visits during prior calendar year (tertiles), n (%) |  |  |  |
| 1 | 4,804 (24.4%) | 5,115 (24.8%) | 0.01 |
| 2 | 6,892 (35.0%) | 7,188 (34.8%) | 0 |
| 3 | 7,611 (38.6%) | 8,009 (38.8%) | 0 |
| Missing | 391 (2.0%) | 348 (1.7%) | 0.02 |
