# Supplementary material for: Use of virtual care near the end of life before and during the COVID-19 pandemic: A population-based cohort study
Source: PLoS One. 2025 Jan 8;20(1):e0313766. doi: 10.1371/journal.pone.0313766 (PMC11709317; doi:10.1371/journal.pone.0313766)
Supplement: S5 Table — (DOCX) [file pone.0313766.s005.docx]

**S5 Table – Comparison of baseline characteristics of people in their last 3 months of life who died in Ontario between 2018 and 2022 who were included in the study to those who were excluded from the study based on their being hospitalized for the entire duration of the study period.**

|  | **Included**  **(n=411,564)** | **Excluded**  **(n=16,845)** | **Standardized Difference** |
| --- | --- | --- | --- |
| Age |  |  |  |
| Median (IQR) | 77 (66-86) | 81 (71-88) | 0.25 |
| 18-29 | 6,202 (1.5%) | 79 (0.5%) | 0.11 |
| 30-39 | 8,807 (2.1%) | 143 (0.8%) | 0.11 |
| 40-49 | 13,736 (3.3%) | 284 (1.7%) | 0.11 |
| 50-59 | 34,910 (8.5%) | 954 (5.7%) | 0.11 |
| 60-69 | 68,142 (16.6%) | 2,220 (13.2%) | 0.1 |
| 70-79 | 97,083 (23.6%) | 3,994 (23.7%) | 0 |
| 80-89 | 115,323 (28.0%) | 5,691 (33.8%) | 0.12 |
| 90+ | 67,361 (16.4%) | 3,480 (20.7%) | 0.11 |
| Female Sex, n (%) | 185,272 (45.0%) | 7,548 (44.8%) | 0 |
| Neighbourhood income quintile, n (%) |  |  |  |
| 1 | 109,070 (26.5%) | 5,063 (30.1%) | 0.08 |
| 2 | 90,997 (22.1%) | 3,778 (22.4%) | 0.01 |
| 3 | 77,353 (18.8%) | 3,012 (17.9%) | 0.02 |
| 4 | 68,141 (16.6%) | 2,375 (14.1%) | 0.07 |
| 5 | 64,414 (15.7%) | 2,414 (14.3%) | 0.04 |
| Ethnicity, n(%) |  |  |  |
| Chinese | 10,663 (2.6%) | 419 (2.5%) | 0.01 |
| South Asian | 8,878 (2.2%) | 269 (1.6%) | 0.04 |
| General Population | 391,874 (95.2%) | 16,153 (95.9%) | 0.03 |
| Rural, n(%) | 52,955 (12.9%) | 1,889 (11.2%) | 0.05 |
| Chronic Conditions, n (%) |  |  |  |
| Cancer | 184,440 (44.8%) | 7,776 (46.2%) | 0.03 |
| Heart failure | 91,955 (22.3%) | 6,036 (35.8%) | 0.3 |
| COPD | 62,529 (15.2%) | 3,051 (18.1%) | 0.08 |
| Dementia | 55,363 (13.5%) | 6,633 (39.4%) | 0.62 |
| Severe liver disease | 5,403 (1.3%) | 329 (2.0%) | 0.05 |
| Diabetes | 133,916 (32.5%) | 7,148 (42.4%) | 0.21 |
| Hypertension | 218,444 (53.1%) | 11,409 (67.7%) | 0.3 |
| End-stage renal disease | 86,116 (20.9%) | 6,437 (38.2%) | 0.39 |
| Stroke | 21,525 (5.2%) | 2,678 (15.9%) | 0.35 |
| Psychotic disorder | 8,179 (2.0%) | 495 (2.9%) | 0.06 |
| Non-psychotic disorder | 98,831 (24.0%) | 4,363 (25.9%) | 0.04 |
| Alcohol and substance use  disorder | 24,582 (6.0%) | 569 (3.4%) | 0.12 |
| Hospital frailty risk score, n (%) |  |  |  |
| 0 | 45,030 (10.9%) | 712 (4.2%) | 0.26 |
| 0.1 - 4.9 | 89,782 (21.8%) | 2,715 (16.1%) | 0.15 |
| 5.0 - 8.9 | 48,041 (11.7%) | 2,600 (15.4%) | 0.11 |
| 9.0 + | 79,282 (19.3%) | 8,458 (50.2%) | 0.69 |
| No prior hospitalizations | 149,429 (36.3%) | 2,360 (14.0%) | 0.53 |
| No. of ED visits not resulting in hospitalization, mean ± SD | 1.12 ± 2.57 | 1.32 ± 2.50 | 0.08 |
| No. of hospitalization episodes, mean ± SD | 0.67 ± 1.20 | 1.16 ± 1.43 | 0.37 |
| Receipt of palliative care in year prior to index, n (%) | 30,752 (7.5%) | 1,002 (5.9%) | 0.06 |
| Designated end-of-life, n (%) | 39,500 (9.6%) | 1,326 (7.9%) | 0.06 |

COPD – Chronic Obstructive Pulmonary Disease; ED – Emergency department
