# Supplementary material for: Use of virtual care near the end of life before and during the COVID-19 pandemic: A population-based cohort study
Source: PLoS One. 2025 Jan 8;20(1):e0313766. doi: 10.1371/journal.pone.0313766 (PMC11709317; doi:10.1371/journal.pone.0313766)
Supplement: S7 Table — (DOCX) [file pone.0313766.s007.docx]

**S7 Table – Dataset Creation and Analysis Plan**

| Project InitiationThis Section must be Completed Prior to Project Dataset(s) Creation | | | | | | |
| --- | --- | --- | --- | --- | --- | --- |
| **Project Title:** | Virtual Palliative Care in Ontario | | | | | |
| **Project TRIM number:** | 2022 0990 564 000 | | | | | |
| **Research Program:** | PCHS | | | | | |
| **Site:** | ICES Central | | | | | |
| **Project Purpose and Objectives:** | *Insert Project Purpose and Objectives as listed in the approved ICES Project PIA* | | | | | |
|  | 1. **Describe** and **identify** characteristics of adult **patients** at the end of life and their **providers** that predict receipt of virtual palliative care 2. **Identify patient** and **provider** factors associated with secular trends in **location of death** (home, hospital) and **rates of acute healthcare use** (emergency department, hospitalization) among **patients** who received virtual palliative care before and after the introduction of specialized virtual fee codes. | | | | | |
| **ICES Project PIA Initial Approval Date:** | *The ICES Employee or agent who is responsible for creating the Project Dataset(s) is responsible for ensuring there is an approved ICES Project PIA and verifying the date of approval prior to creating the Project Dataset(s)* | | | | | |
|  | 2021-Jul-29 | | | | | |
| **Principal Investigator (PI):** | Kieran Quinn | | | | | |
| **Check the applicable box if the PI is an ICES Student/Trainee** | ICES Student  ICES Fellow  ICES Post-Doctoral Trainee  Visiting Scholar | | | | | |
| **Responsible ICES Scientist:** | *Name the Responsible ICES Scientist if the PI is not a Full Status ICES Scientist* | | | | | |
|  | Chaim Bell | | | | | |
| **Project Team Member(s) Responsible for Project Dataset Creation and/or Statistical Analysis and date joined (list all):** | *All person(s) (ICES Analyst, Appointed Analyst, Analytic Epidemiologist, PI, and/or Student) responsible for creating the Project Dataset(s) and/or statistical analysis on the Research Analytics Environment (RAE) and/or Data Safe Haven (DSH) and the date they joined the project must be recorded* | | | | | |
|  | TBD | | | yyyy-mon-dd | | |
| **Project Team Member(s) who will request RAE and/or DSH folder access (list all):** | *List the project team member responsible for dataset creation who will request access for all members requiring RAE and/or DSH project folder access (e.g. analyst, methodologist, student, etc).* | | | | | |
|  | TBD | | yyyy-mon-dd | | |  |
| **Other ICES Project Team Members and date joined (list all):** | *All other Research Project Team Members (e.g., Research Administrative Assistants, Research Assistants, Project Managers, Epidemiologists) and the date they joined the project must be recorded* | | | | | |
|  |  | | |  | | |
| **Confirmation that DCP is consistent with Project Objectives:** | *The following individuals must confirm that the ICES Data provided for in this DCP is relevant (e.g., with respect to cohort, timeframe, and variables) and required to achieve the purpose(s) stated in the ICES Project PIA prior to initial Project Dataset creation: 1) PI; 2) Responsible ICES Scientist if the PI is not a Full Status ICES Scientist, or a second ICES Scientist or the Scientific Program Lead if the PI is creating both the DCP and the Project Dataset[s]; 3) ICES Research and Analysis Staff creating the DCP; and 4) ICES Analytic Staff (ICES Employee or agent responsible for creating the Project Dataset[s]). Documentation of this confirmation may be delegated either verbally or via e-mail.* | | | | | |
|  | ***Principal Investigator*** |  | | | 2021-SEPT-15 | |
|  | ***Responsible ICES Scientist or Second ICES Scientist/Lead*** |  | | 2021-SEPT-15 | | |
|  | ***ICES Research and Analysis Staff Creating the DCP*** |  | | yyyy-mon-dd | | |
|  | ***ICES Analytic Staff*** |  | | yyyy-mon-dd | | |
| **Designated ICES Research and Analysis Staff accountable for Project Documentation:** | *The person named (ICES staff) is accountable for ensuring that the approved ICES Project PIA, ICES Project PIA Amendments, and DCP are saved on the T Drive, ensuring ICES Project PIA Amendments are submitted as required, ensuring DCP Amendments are documented, and sharing the final DCP with the PI/Responsible ICES Scientist at project completion* | | | | | |
|  | Farah Eva Saxena | | | | | |

| **DCP Creation Date and Author:** | *Date DCP was finalized prior to Project Dataset(s) creation* | *Name of person who created the DCP* |
| --- | --- | --- |
|  | ***Date*** | ***Name*** |
|  | 2021-Aug-23 | Farah Eva Saxena |

| ICES DataThis Section must be Completed Prior to Project Dataset(s) Creation | |
| --- | --- |
| *The ICES Employee or agent who is responsible for creating the Project Dataset(s) must ensure that this list includes only data listed in the ICES Project PIA*  *Changes to this list after initial ICES Project PIA approval require an ICES Project PIA Amendment* | *Mandatory for all datasets that are available by individual year* |
| ***General Use Datasets – Health Services*** | ***Years (where applicable)*** |
| CCRS | Most update |
| CIHI DAD | 2002-2021 |
| CIHI SDS | 2002-2021 |
| CONTACT | 2002-2022 |
| CPRO | Most update |
| ERCLAIM | 2002-2022 |
| HCD | Most update |
| HCDMOH | Most update |
| NACRS | 2002-2021 |
| NRS | Most update |
| ODB | 2002-2022 |
| OHCAS | 2002-2004 |
| OHIP | 2002-2022 |
| OMHRS | Most update |
| RAIHC | Most update |
| RAIHCMOH | Most update |
| ***General Use Datasets – Care Providers*** |  |
| CPDB | Most update |
| IPDB | Most update |
| ***General Use Datasets – Population*** |  |
| RPDB | Most update |
| CENSUS | 2001, 2006, 2011, 2016 |
| POP | 2002-2022 |
| ***General Use Datasets – Coding/Geography*** |  |
| DIN |  |
| REF |  |
| LHIN |  |
| PCCF |  |
| ***General Use Datasets - Facilities*** |  |
| INST |  |
| ***General Use Datasets - Other*** |  |
| ASTHMA |  |
| CAPE |  |
| CHF |  |
| COPD |  |
| HYPER |  |
| ODD |  |
| OMID |  |
| ***Controlled Use Datasets*** |  |
| CIC | Most update |
| See list |  |
| ***Other Datasets (including PSD and PDC data)*** |  |
| RAICA | Most update |
|  |  |
| NMS | Most update |
| ESAS | Most update |
| ETHNIC | Most update |

| Project Amendments and Reconciliation | | | |
| --- | --- | --- | --- |
| **ICES Project PIA Amendment History (add additional rows as needed):** | *Privacy approval date* | *Person who submitted amendment* | *Note that any changes to the list of ICES Data or Project Objectives require an ICES Project PIA Amendment* |
|  | ***Date*** | ***Name*** | ***Amendment*** |
|  | 2021-Sept-03 | Farah Saxena | Add ETHNIC dataset |
| **DCP Amendment History (add additional rows as needed):** | *Date DCP amended* | *Person who made the DCP amendment* | *Note that any DCP amendments involving changes to the list of ICES Data or Project Objectives require an ICES Project PIA Amendment* |
|  | ***Date*** | ***Name*** | ***Amendment*** |
|  | 2021-Aug-31  2021-Sept-03  2021-Sept-07  2021-Sept-08  2021-Sept-13  2021-Sept-14  2021-Sept-30  2021-Oct-05  2021-Oct-12  2021-Oct-18  2021-Oct-19  2021-Oct-20  2021-Nov-25  2021-Dec-14  2022-Jan-17  2022-Feb-28  2024-Jan-10  2024-Jan-11 | Kieran Quinn  Farah Saxena  Kieran Quinn  Farah Saxena  Kieran Quinn  Farah Saxena  Farah Saxena  Farah Saxena  Farah Saxena  Farah Saxena  Farah Saxena  Farah Saxena  Farah Saxena  Farah Saxena  Hannah Chung  Hannah Chung  Hannah Chung  Kieran Quinn | Updates to DCP after reviewing first draft  Updates to DCP and tables based on Kieran’s feedback, still finalizing DCP  Second iteration feedback on DCP  Minor updates to DCP and tables  Minor updates to DCP following Jun’s review  Minor updates to DCP and tables  Addition of LHIN into analyses  Updates to DCP based on kick off meeting  Updating comorbidities definitions  Better defining virtual palliative care covariate and mental health conditions  Adding sensitivity analysis for patients whose index and death date cross over at March 14  Updating physician assignment criteria  Updated physician assignment criteria and added new continuity of care secondary outcome var  Updates to AIM 1 models  Updated exclusions based on physician descriptives (see email for Kieran Quinn on Tuesday December 21, 2021)  Clarificaiton on Tables 14, 17 & 18 – different cohort where exposure is receipt of virtual palliative care between death and 90-days before death (index date of main cohort) and secondary outcomes (healthcare utilization and location of death)  Extend accrual period to November 30, 2022  Add ONMARG quintiles  For variables with “missing” category, include as separate category in model (i.e., no longer doing case series modelling). Only those with missing physician will get excluded.  Added outcome measure of % receiving specific virtual PallC codes between July 1, 2021 and Nov 30, 2022  Added reporting of baseline characteristics of people excluded from the study due to hospitalization during the entireity of the study period |
| **Date Programs/DCP reconciled** |  | | |
|  | yyyy-mon-dd | | |

| Project Cohort | | |
| --- | --- | --- |
| **Study Design** | Decedent Cohort study  Matched cohort study  Case-control study  Cross-sectional study  Other (specify): | |
| **Index Event / Inclusion Criteria**  *(please ensure index event / inclusion criteria are specified with data sources, variables, study period and values or codes)* | All adults aged 18 or older who died in Ontario between January 25, 2018 and November 30, 2022 using RPDB  **Index date:** 90 days prior to death  *Some individuals will have an index date pre-March 14 and death date post-March 14 🡪 Flag these individuals, so we know how many patients span the time frame in this manner and also flag how much time after March 14 that they died (those who died later may affect the results more)*   - 22,208 patients crossed over March 14, 2020 in this manner, which looks like a normal distribution accounting for 7% of the final cohort - This group of individuals were allocated 50:50 to the pre- and post-March 14, 2020 based on timing of index to March 14, 2020 (patients were ranked by their interval and split evenly 50:50) | |
| **Estimated Size of Cohort** | Approximately 400,000 | |
| **Exclusions** *(in order)  (common exclusions are listed in grey italics for consideration)* | *Step* | Description |
|  | 1 | Invalid IKN |
|  | 3 | Invalid/missing data on AGE or SEX at index date |
|  | 4 | Age < 18 years at index date or >105 |
|  | 2 | Not Ontario resident (substr(prcddablk, 1, 2) not = ‘35’) at index date |
|  | 3 | Invalid/missing data on AGE or SEX at index date |
|  | 4 | Not eligible for OHIP for ≥3 months in the year prior to index date |
|  | 5 | Residing in Long-Term Care at index date (CCRS-LTC any assessment, or ODB LTC flag = "1" (indicating prescription in LTC), or OHIP with "W" fee code AND linked INST.MNS insttype IN ("NH","HF", “TM”, “LT”) using 2 year look-back) |
|  | 6 | DOLC > 10 years |
|  | 7 | If institutionalized for the entire follow-up period (days_in_inst ≥ 90) |
|  | 8 | Exclude patients who are not assigned to a physician |

| Project Time Frame Definitions | | |
| --- | --- | --- |
| Look-back Window  Observation Window  (in which to look for outcomes)  **Index Event Date**  Accrual Window  Max Follow-up Date | |  |
| **Accrual Start/End Dates** | January 25, 2018 to November 30, 2022 |  |
| **Max Follow-up Date** | November 30, 2022 |  |
| **When does observation window terminate?** | November 30, 2022 |  |
| **Lookback Window(s)**  *(please ensure lookback windows are defined with start and end dates and in relation to the index event date)* | 5 years |  |

| Variable Definitions (add additional rows as needed) *A few key points to keep in mind:*   1. *Please ensure codes, data sources, diagnosis types and lookback periods (if applicable) are provided for all definitions listed below and that codes are provided in Excel format. If borrowing codes from another project, please list all the codes here* 2. *There are maximum number of digits that can be specified using ICES data (ICD 9 CA codes are up to maximum of 4 digits, ICD 10 CA codes are 6 digits, OHIP diagnosis codes are 3 digits)* | | |
| --- | --- | --- |
| **Main Exposure or Risk Factor** | **Primary exposure variable:**  **March 14, 2020 (introduction of new virtual care feecodes)**  **We will measure the use of virtual palliative care (Y/N) before and after March 14, 2020*   1. Virtual codes are provided in the Appendix outlining feecodes used during the pre-pandemic period and new ones introduced on March 14, 2020    1. Irrespective of when the visit was during the study period (2018 to 2021), determine whether the visit was a home-based or virtual palliative care visit based on the following:       - For visits in the year before index date of the individual, generic* virtual care codes must be paired with a home-based palliative care feecode on the same date to be considered virtual palliative care visit.       - Between index date and death, generic virtual care codes alone were assumed to be palliative (and even if paired with home-based, I considered them as virtual)         - *Generic virtual codes = non-G511A (pre) and non-K092A/K093A/K094A/K095A         - G511 can occur at any time period. However, the other specific codes are only pre/post March 14th   **Predictor variables:**  **Patient characteristics**   1. Age (Mean (SD), median (IQR), and decades 18 – 29, 30 – 39, 40 – 49, etc.) 2. Sex 3. Income quintile (1,2,3,4,5,missing) 4. ONMARG Quintiles (1,2,3,4,5,missing)    1. Household and Dwellings    2. Material Resources    3. Age and Labour Force    4. Racialized and Newcomer Population 5. Ethnicity (ETHNIC)    1. South Asian    2. Chinese    3. General Population    4. Missing 6. Rurality (urban, rural, missing; from StatsCan definition) 7. Patient’s LHIN:  - Erie Saint Clair - South West - Waterloo Wellington - Hamilton Niagara Haldimand Brant - Central West - Mississauga Halton - Toronto Central - Central - Central East - South East - Champlain - North Simcoe Muskoka - North East - North West   (*NOTE: Reference group for modelling will be the LHIN with the lowest proportion of patients who received virtual palliative care.)   1. Comorbidities (use 5-year lookback from index)    1. Heart failure    2. COPD    3. Diabetes    4. Dementia    5. Hypertension    6. Cancer    7. End-stage renal disease    8. Stroke    9. Decompensated cirrhosis in the past 5 years (using validation study <https://journals.plos.org/plosone/article?id=10.1371/journal.pone.0201120>, not part of %hsprn_mmb):       - The most sensitive algorithm for decompensated cirrhosis was one cirrhosis code with any of: a hospital diagnostic code, death code, or procedure code for decompensation (see Appendix for codes)    10. Psychotic disorder    11. Non-psychotic disorder    12. Alcohol and substance use disorder        - For the three MHA-related conditions above, please consider 2 outpatient or 1 hospital-based (ED and DAD/OMHRS) diagnosis in the past 5 years as indicative of having the condition. Please refer to the ICES MHA team’s methodologies for defining MHA outpatient visits and MHA ED and hospitalizations, which can be found in the ICES        - Move the OHIP 296 feecode from psychotic category to non-psychotic and exclude DAD/OMHRS categories DSH (deliberate self harm) and NONCLA (developmental disorders, autism, etc.) as they do not clearly fall into any of our mental health categories of interest and do not map one-to-one with OHIP categories 2. Hospital frailty risk score: 0, 0.1-4.9, 5-8.9, 9+, missing (use macro %hospfrailty) – based on last 5-years of hospitalization data 3. Healthcare system use in the 365 days prior to study index date    1. Number of unique prescription drugs based on DINs (Count once per DIN per IKN per prescription day). Do not exclude any DINs to be consistent with previous studies.    2. Number of unique emergency department episodes not resulting in hospitalization       - Exclude scheduled visits       - Exclude transfers from another ED       - Exclude duplicate ED visits       - Exclude ED visits where patient left before being seen       - Exclude ED visits that lead to hospitalizations    3. Number of unique hospitalization episodes (both DAD and OMHRS episodes, not individual hospital admissions)    4. Receipt of home or virtual palliative care in the 1 year prior to index       - Any pandemic or pre-pandemic virtual care code must be concurrently billed with a home-based palliative care feecode. For example, a virtual mental health visit is not palliative if billed outside the 3 months prior to death, unless billed with a specific palliative care code or concurrently biled with a home-based palliative care feecode. – *used as covariate in models*       - Patients previously receiving in-person home-based palliative care (had ≥2 home-based palliative care fee codes billed in the 1 year prior to index date where dates of feecodes are ≤ 90 days apart) – *specific definition for subgroup analyses* 4. Receiving long-stay homecare (use 2-year lookback for completion of RAI).    1. Designated end of life homecare    2. HCD is only updated to Sept 2023, please flag anyone we don’t have information for after this time period as missing   **Provider-level characteristics (from CPDB and supplemented with information from IPDB where not available, unless otherwise specified)**  *The underlying question: who is most responsible for their EOL care and how does this impact their likelihood of receiving virtual EOL care?*  *A provider should only appear once in each pre- or post-period, so that physicians are not over-represented*  *For those who don’t have any palliative care codes claimed before they died, first determine how many people are affected in this way, then use the following hierarchy to assign the physician:*   - 1. *The physician with the most number of unique visits to the patient during follow-up (limit this to home, office, and phone visits to reduce noise that hospital and ED OHIP billings might contribute)*      - *If >1 physician, then select physician who billed palliative care*      - *If >1 palliative care physnum, then pick by random selection*      - *If no palliatve care physician, then pick by random selection*   2. *If no follow-up visits, then use the rostered/virtually rostered approach to assign a family doctor to these individuals*   3. *If no follow-up and not rostered, then use most recent palliative care physnum pre-index*   4. *If no follow-up and not rostered and no pre-index palliative care physnum, then use physician who billed (outpatient visits) the most pre-index*      - *Exclude physicians from assignment if they have the following specialties:*   5. *Exclude physicians with ≤5 years of practice (attempt to reassign patients that were initially assigned to these physicians and then drop the remaining unassigned patients)*   For the **main study**, use physician characteristics from the calendar year prior to the paired patient’s index date for models (see email from November 15, 2021)  *Physician characteristics shoud be derived from the midpoint of the period that physician is assigned to (2018 for first period, 2020 for second period)*   - 1. Age   2. Sex   3. Practice location (rural vs. urban)   4. Years in practice (provide distribution in percentiles to create categories from this)   5. Practice specialty      1. GP      2. Specialist   6. Status as a palliative care specialist (OHIP)      - Providers who bill >10% of their total OHIP fee codes in a given year are deemed palliative care specialists   7. Education (Canadian vs. international medical graduate)   8. Volume of fee claims per year (based on calendar year): OHIP      - Unique visits (includes all visits, including those outside our cohort)        - Proportion of visits the physician did for the calendar year        - Proportion of visits the physician did for thecalendar year that was any palliative care        - Proportion of visits the physician did for the calendar year that was virtual palliative care only        - Proportion of visits the physician did for the calendar year that was in-person palliative care only      - Unique patients (measurememt of intensity of care)        - Median number of unique patients seen per physician        - Median number of unique visits per unique patient during follow up |  |
| **Primary Outcome Definition** | 1. **Receipt of any virtual care within the last 3 months of life [Y/N]**    1. Receipt of virtual palliative care (among decedents who died between July 1, 2021 to Nov 30, 2022 when specific virtual palliative care fee codes were introduced – K092-K095)       1. numerator = number of people with ≥1 virtual PallC code; denominator = number of people with ≥1 of any virtual code |  |
| **Secondary Outcome Definition(s)** | 1. **Number of home-based palliative care visits during follow-up from any provider** 2. **Number of virtual palliative care visits during follow-up from any provider** 3. **Number of unique physicians (mean/SD, median/IQR) that provided any palliative care (using OHIP codes in Appendix) to each patient from:**    1. Index date to death.    2. 1 year prior to index date. 4. **Number of unique physicians (mean/SD, median/IQR) that provided virtual palliative care (using OHIP codes in Appendix) to each patient from:**    1. Index date to death.    2. 1 year prior to index date. 5. **Palliative care delivery models (% of decendents receiving)**   **Virtual care and home-based OHIP feecodes can be found in the Appendix*   - 1. Exclusively virtual (**Patient had only virtual fee codes billed during follow-up)*   2. Exclusively home-based (**Patient had only home-based fee codes billed during follow-up)*   3. Mixed virtual and home-based *(*At least one virtual care code and one home-based code present during follow-up period)*  1. % of assigned physicians who also billed a virtual feecode for the patient (if the patient received virtual care |  |
| **Baseline Characteristics** | Patient and provider-level charactersitics as listed above |  |
| **Other Variables** |  |  |

| Analysis Plan and Dummy Tables (expand/modify as needed) *(please ensure the analysis plan is outlined with dummy tables (can be a separate document)*  *and clear specification of exposures / outcomes / covariates for each model)* | | |
| --- | --- | --- |
| **Descriptive Tables (insert or append dummy tables), e.g.:** | | |
| **Table 1. Baseline characteristics of patients,providers and patient LHIN at index date and comparing baseline characteristics before and after March 14, 2020.**  *****Report these characteristics for people excluded from study due to hospitalization during entirety of final 3 months of life, create second table separated by time period (pre and post March 14)** | | |
| **Table 2. Provider characteristics by physician anchor date.** | | |
| **Table 3. The effect of health policy intervention on the use of virtual EOL care after adjustment for potential differences in patient characteristics** | | |
| **Table 4. Association of patient- and physician characteristics with the use of EOL virtual palliative care in pre (Table 4a)- and post (Table 4b)- March 14, 2020 time periods** | | |
| **Table 5. Association of patient characteristics with the time period corresponding to the use of EOL virtual palliative care (post- compared to pre-March 14, 2020) among those who received EOL virtual palliative care.** *(Restricted to individuals who receive virtual palliative care during follow-up)* | | |
| **Table 6. Receipt of any virtual palliative care in the overall study population and subgroups of interest stratified by time period.** *(Restricted to individuals who receive virtual palliative care during follow-up)* | | |
|  | | |
| **Table 8. Delivery of any virtual palliative care across patient characteristics and patient LHIN stratified by time period.** *(Restricted to individuals who receive virtual palliative care during follow-up)* | | |
| **Table 9. Comparing secondary outcomes of interest between periods for receiving any virtual palliative care.** *(Restricted to individuals who receive virtual palliative care during follow-up)* | | |
|  | | |
| **Table 10. Rates of acute health care use and location of death among patients who received any virtual palliative care before and after the introduction of specific virtual palliative care feecodes.** *(Restricted to individuals who receive virtual palliative care during follow-up)* | | |
| **Table 11a. Patient and provider-level characteristics associated with healthcare use before March 14, 2020.** | | |
| **Table 11b. Patient and provider-level characteristics associated with healthcare use on or after March 14, 2020.** | | |
| **Table 12a. Patient and provider-level characteristics associated with location of death stratified by time period (before March 14, 2020)** | | |
| **Table 12b. Patient and provider-level characteristics associated with location of death stratified by time period (on/after March 14, 2020)** | | |
| **Table 13. Healthcare use in the overall study population and subgroups of interest stratified by time period.** | | |
|  | | |
|  | | |
| **Table 15. Location of death in the overall study population and subgroups of interest stratified by time period.** | | |
|  | | |
|  | | |
| **Table 17. Association between pre/post March 14^th^ (exposure) and healthcare use (outcome) in main cohort and among subgroups of interest.** | | |
| **Table 18 –Association between pre/post March 14^th^ (exposure) and location of death (outcome) in main cohort and among subgroups of interest.** | | |
| **Figure 1. Forest plot displaying effect estimates for receipt of virtual palliative care in the overall population and subgroups of interest** | | |
| **Figure 2. Forest plot displaying effect estimates for healthcare use and location of death in the overall population and subgroups of interest** | | |
| **Statistical Model(s) for AIM 1** | | |
| **Type of model** | Multi-level moodified Poisson Regression (cluster by assigned physician) | |
| **Primary independent variable** | Introduction of specialized virtual feecodes (analysis will be conducted ont he whole cohort, on sub-groups of those ‘before March 14, 2020’ and ‘on or after March 14, 2020 to November 30, 2022’, and another subgroup of those who received the outcome)  NOTE: For tables stratified by time period (pre/post-March 14, 2020), independent variable(s) are each of the patient- and provider-level characteristics | |
| **Dependent variable** | Receipt of any virtual palliative care (Y/N) | |
| **Covariates** | Patient- and provider-level characteristics | |
| **Analyses details** |  | |
| **Statistical Model(s) for AIM 2** | | |
| **Type of model** | Multivariable logistic/Poisson regression | |
| **Primary independent variable** | Introduction of specialized virtual feecodes (analysis will be stratified by ‘before March 14, 2020’ and ‘on or after March 14, 2020 to January 24, 2022’)  **Secondary independent variable:** Pandemic time periods of pre-pandemic, 1^st^ pre-pandemic wave, 1^st^ post-pandemic wave, 2^nd^ wave, 3^rd^ wave, 4^th^ wave as defined above | |
| **Dependent variable** | Acute care visits (ED visit; Hospitalization) and location of death (Hospital vs. out-of-hospital) | |
| **Covariates** | Patient and provider-level characteristics (first provider billing any palliative care following index date) | |
| **Analyses details** |  |  |

| Quality Assurance Activities | | | |
| --- | --- | --- | --- |
| **RAE/DSH Directory of SAS Programs** |  | | |
| **RAE/DSH Directory of Final Dataset(s)** | *The* *final analytic dataset for each cohort includes all the data required to create the baseline tables and run all the models. It should include all covariates for all models such as patient risk factors, hospital characteristics, physician characteristics, exposure measures (continuous, categorical) and outcomes. It should include covariates that were considered but didn’t make the final cut. This would permit an analyst to easily re-run the models in the future.* | | |
|  |  | | |
| **RAE/DSH README file available:** Yes No | | | |
| **Date results of quality assurance tools for final dataset shared with project team (where applicable):** | | |  |
|  | | **%assign** | yyyy-mon-dd |
|  | | **%evolution** | yyyy-mon-dd |
|  | | **%dinexplore** | yyyy-mon-dd |
|  | | **%track / %exclude** | yyyy-mon-dd |
|  | | **%codebook** | yyyy-mon-dd |
| **Additional comments:** | |  | |

**APPENDIX**

**Receipt of Virtual Palliative Care**

Use the following codes to measure delivery of virtual palliative care:

**Pre-Pandemic Fee Codes**

B099A Tracking Code

B100A First Telemedicine Patient Encounter premium

B101A First Cancelled/Missed Telemedicine Patient Encounter premium

B102A First Technical Difficulties Abandoned Patient Encounter premium

B200A Subsequent Telemedicine Patient Encounter premium

B201A Subsequent Missed/Cancelled Telemedicine Patient Encounter premium

B202A Subsequent Technical Difficulties Abandoned Patient Encounter premium

G511A Telephone management regarding a patient receiving palliative care at home (CAN BE BILLED AT ANY TIME PERIOD)

**Pandemic Fee Codes**

K080A Minor assessment of a patient by telephone or video or advice or information

by telephone or video to a patient’s representative regarding health maintenance, diagnosis, treatment and/or prognosis.

K081A *a.* Intermediate assessment of a patient by telephone or video, or advice or

information by telephone or video to a patient’s representative regarding health maintenance, diagnosis, treatment and/or prognosis, if the service lasts a minimum of 10 minutes; or *b.* Psychotherapy, psychiatric or primary mental health care, counselling or interview conducted by telephone or video, if the service lasts a minimum of 10 minutes.

K082A Psychotherapy, psychiatric or primary mental health care, counselling or

interview conducted by telephone or video per unit (unit means half hour or major part thereof).

K083A Specialist Consultations and Visits by telephone or video.

B203A Synchronous video visits with a patient in the home or another location of their

choice (i.e. the patient is not at a patient host site).

K092A Virtual Palliative Care Consultation-Telephone

K093A Virtual Palliative Care Consultation-Video

K094A Virtual Palliative Care Support-Telephone

K095A Virtual Palliative Care Support-Video

**Virtual palliative care fee codes were introduced for use on July 1^st^, 2021*

**Receipt of Home-Based Palliative Care**

1. A900 with (B966, B998, B997): Complex house call assessment
2. A901 with (B966, B998, B997): House call assessment
3. A945 with any B code: Special palliative care consultation
4. K023 with A900 A901 or any B code: Palliative care support
5. K015 with A900 A901 or any B code: Counselling of relatives ­on behalf of catastrophically or terminally ill patient
6. B966: Palliative care home visit; travel premium – weekdays daytime
7. B998: Palliative care home visit; special visit premium – weekdays daytime, first-person seen
8. B997: Palliative care home visit; special visit premium – nights, first-person seen
9. A900 A901 B960 B961 B962 B963 B964 B986 B987 B988 B990 B992 B993 B994 B996 within the last 3 months prior to death


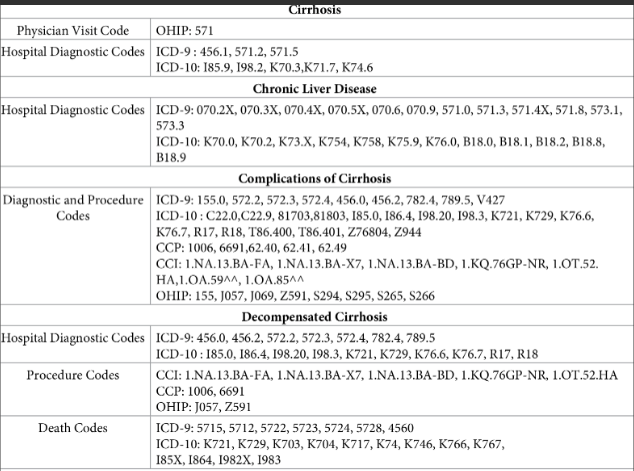


**List of physician specialties to exclude from outpatient visits when assigning physicians:**

| ADOLESCENT MEDICINE |
| --- |
| ANATOMICAL PATHOLOGY |
| CHILD & ADOLESCENT PSYCHIATRY |
| DEVELOPMENTAL PEDIATRICS |
| DIAGNOSTIC RADIOLOGY |
| FELLOW |
| FORENSIC PATHOLOGY |
| GENERAL PATHOLOGY |
| LAB MEDICINE |
| MATERNAL FETAL MEDICINE |
| MEDICAL BIOCHEMISTRY |
| MEDICAL MICROBIOLOGY |
| NEONATAL/PERINATAL MEDICINE |
| NEUROPATHOLOGY |
| NUCLEAR MEDICINE |
| OUT OF COUNTRY |
| OUT OF PROVINCE |
| PEDIATRIC CARDIOLOGY |
| PEDIATRIC CLINICAL IMMUNOLOGY |
| PEDIATRIC CRITICAL CARE |
| PEDIATRIC EMERGENCY MEDICINE |
| PEDIATRIC ENDOCRINOLOGY |
| PEDIATRIC GASTROENTEROLOGY |
| PEDIATRIC HEMATOLOGY |
| PEDIATRIC INFECTIOUS DISEASES |
| PEDIATRIC NEPHROLOGY |
| PEDIATRIC NEUROLOGY |
| PEDIATRIC RADIOLOGY |
| PEDIATRIC RESPIROLOGY |
| PEDIATRIC RHEUMATOLOGY |
| PEDIATRIC SURGERY |
| PEDIATRICS |
